# Genetic and Epigenetic Regulation of TOX3 Expression in Breast Cancer

**DOI:** 10.1371/journal.pone.0165559

**Published:** 2016-11-02

**Authors:** Yoo-Jeong Han, Jing Zhang, Yonglan Zheng, Dezheng Huo, Olufunmilayo I. Olopade

**Affiliations:** 1 Center for Clinical Cancer Genetics and Global Health; and Section of Hematology and Oncology, Department of Medicine, University of Chicago, Chicago, IL 60637, United States of America; 2 Department of Public Health Sciences, University of Chicago, Chicago, IL 60637, United States of America; Sapporo Ika Daigaku, JAPAN

## Abstract

Genome wide association studies (GWAS) have identified low penetrance and high frequency single nucleotide polymorphisms (SNPs) that contribute to genetic susceptibility of breast cancer. The SNPs at 16q12, close to the *TOX3* and *CASC16* genes, represent one of the susceptibility loci identified by GWAS, showing strong evidence for breast cancer association across various populations. To examine molecular mechanisms of *TOX3* regulation in breast cancer, we investigated both genetic and epigenetic factors using cell lines and datasets derived from primary breast tumors available through The Cancer Genome Atlas (TCGA). TOX3 expression is highly up-regulated in luminal subtype tumors compared to normal breast tissues or basal-like tumors. Expression quantitative trait loci (eQTL) analyses revealed significant associations of rs3803662 and rs4784227 genotypes with TOX3 expression in breast tumors. Bisulfite sequencing of four CpG islands in the *TOX3* promoter showed a clear difference between luminal and basal-like cancer cell lines. 5-Aza-2’-deoxycytidine treatment of a basal-like cancer cell line increased expression of TOX3. TCGA dataset verified significantly lower levels of methylation of the promoter in luminal breast tumors with an inverse correlation between methylation and expression of TOX3. Methylation QTL (mQTL) analyses showed a weak or no correlation of rs3803662 or rs4784227 with TOX3 promoter methylation in breast tumors, indicating an independent relationship between the genetic and epigenetic events. These data suggest a complex system of *TOX3* regulation in breast tumors, driven by germline variants and somatic epigenetic modifications in a subtype specific manner.

## Introduction

Genetic factors play important roles in the etiology of breast cancer. Multiple breast cancer susceptibility genes such as *BRCA1* and *BRCA2* with high penetrant disease-associated mutations have been shown to segregate in families with breast cancer by linkage studies [1]. In addition, Genome Wide Association Studies (GWAS) have identified low penetrance, high frequency single nucleotide polymorphisms (SNPs), revealing more than 90 chromosomal regions that contribute to genetic susceptibility [2,3,4,5,6]. The SNP rs3803662 at 16q12 is one of the susceptibility loci identified by GWAS, for which the minor allele conferred increased risk of breast cancer in women of European ancestry [7]. This finding was also observed in women of Asian descent [8,9] with SNP rs4784227 at 16q12 as an additional risk variant for breast cancer in Asian populations [10]. Evaluation and fine mapping of the susceptibility loci in African Americans identified significant associations with markers at 16q12 SNPs that are independent of the index signal (rs3803662) but represent novel risk variants (rs3104793, rs3104788, rs3104778) with a perfect proxy for rs3112572 [11,12,13,14]. Thus data from diverse populations provide strong evidence for breast cancer association within SNPs at 16q12.

Revealing the molecular bases for the observed associations of SNPs and cancer risks remains challenging because the risk variants map predominantly to non-coding regions and the linked markers merely serve as indicators that a causal variant is present nearby. The 16q12 SNPs reside at introns of a non-protein coding hypothetical gene (*LOC643714)*, which was recently named cancer-susceptibility candidate 16 (*CASC16*). *TOX3* is located ~5 kb downstream of rs3803622 and ~50 kb downstream of rs3104788. Because enhancers are long-range cis-regulatory elements and function over up to mega base-long genomic distances to regulate the expression patterns of their target gene(s), it has been suggested that risk variants control gene expression by altering the activity of enhancers [15]. For example, *in vitro* experimentation demonstrated that the SNP rs4784227, located 18.4 kb upstream of the *TOX3* gene, altered *TOX*3 gene expression by disrupting enhancer function through FOXA1 affinity modulation [16].

TOX3 is a nuclear protein containing a nuclear localization signal and a high mobility group-box domain that can modify chromatin structure. TOX3 binds to the BRCA1 promoter and negatively regulates BRCA1 expression [17]. Ectopic expression of TOX3 increased breast cancer cell proliferation, migration, and survival after exposure to apoptotic stimuli and were associated with tumor progression in a mouse model of breast cancer. *TOX3* is often amplified and overexpressed in breast tumors, particularly in advanced breast tumors [17]. *TOX3* mutations were also found in breast tumors with an overall mutation frequency of 4.5% [18]. Patients with ER positive tumors and high levels of TOX3 mRNA had shorter overall- and distant metastasis free-survival, an effect mostly attributable to patients with luminal B tumors [19].

CpG islands are short sequences of genomic DNA with the length of 0.5 to several kb [20] in which the frequency of the linear 5′-CpG-3′ sequence is higher than at other regions of the gene [21,22]. Aberrant methylation of CpG islands resulting in gene silencing or overexpression of genes has been associated with development and progression of cancers. In this study, we tested if overexpression of *TOX3* is due to epigenetic factors that regulate *TOX3/CASC16* expression in breast cancer cells. We identified hypomethylation of the *TOX3* promoter as a significant contributor of *TOX3* upregulation in luminal subtype breast cancer. We also found a significant association of TOX3 expression with rs3803662 and rs4784227, a risk variant identified in European and Asian women, respectively. These results suggest that both epigenetic and genetic factors contribute to the increased expression of TOX3 in luminal cancer. Thus, our data support a plausible molecular mechanism integrating epigenetic modifications of the *TOX3* promoter and allele specific expression of SNPs in aggressive behavior of luminal breast tumors with high TOX3 expression.

## Results

### Subtype-specific expression of *TOX3/CASC16* in breast cancer cells and tumors

**Fig 1:**
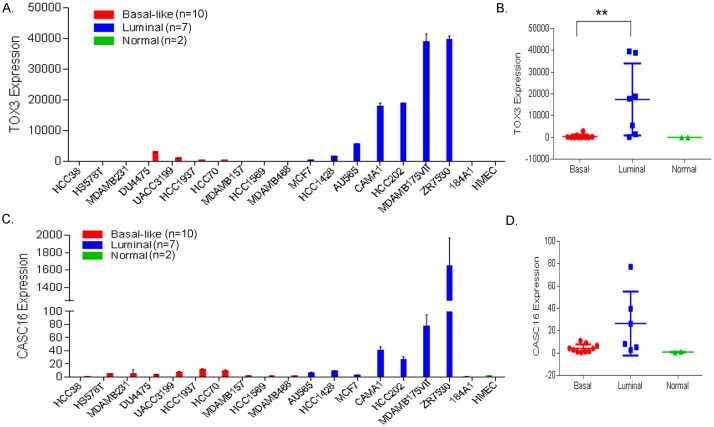
Increased expression of TOX3 and CASC16 in luminal breast cancer cell lines. (A and C) TOX3 and CASC16 expression was assessed in breast cancer cell lines using qRT-PCR relative to expression in normal breast epithelial cell line HMEC (RQ, relative quantity). (B and D) Expression of TOX3 in luminal cell lines (blue) is significantly higher than expression in basal-like cells (red) and non-malignant breast epithelial cells (green) (**p<0.01). CASC16 also exhibits a similar expression pattern with higher expression in luminal cells but fails to reach statistical significance.

The chromosomal region 16q12.1–12.2 contains two genes, a protein-coding gene (*TOX3)* and a long non-coding RNA (*CASC16/LOC643714)*, which generates a long intergenic non-protein coding RNA 918 (Linc00918, RefSeq NR033920.1). We surveyed TOX3 expression in 19 breast cell lines with different molecular subtypes, including basal-like (n = 10) and luminal subtype cells (n = 7) as well as non-malignant mammary cells (n = 2), human mammary epithelial primary cells (HMEC) and immortalized mammary cells (184A1). Comparison of TOX3 expression in these cell lines revealed greater expression of TOX3 in luminal subtype cells compared to normal breast and basal-like subtype cell lines (Fig 1A and 1B). The TOX3 expression levels vary within luminal subtypes, ranging from a minimum of 172.7±13.4-fold increase (MCF-7 cells) to a maximum of 39,526±1,369-fold increase (ZR-75-30 cells) compared to HMEC. LincRNA 918 from *CASC16* exhibits a similar expression pattern with higher expression in luminal subtype cells compared to basal-like and normal breast cell lines (Fig 1C and 1D).

**Fig 2:**
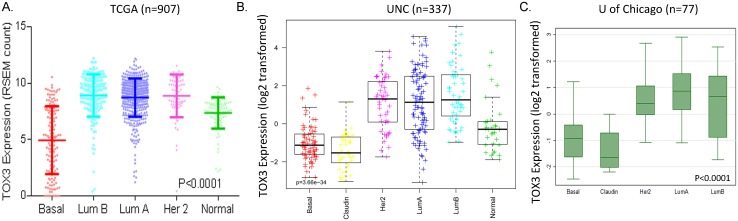
Subtype-specific expression of TOX3 in breast tumors. RNA sequencing (TCGA) and microarray results (UNC and U of Chicago) of TOX3 expression in breast tumors showed a significant difference in TOX3 expression among breast cancer subtypes, with TOX3 expression higher in luminal A, luminal B and Her 2 amplified tumors compared to normal breast epithelial tissues and basal-like tumors. (A) TCGA dataset (p<0.0001), RNA-seq data presented by RSEM (RNA-Seq by Expectation Maximization) normalized counts; (B) the University of North Carolina (UNC) dataset (p = 3.66e-34), microarray data presented by log2 transformed values; (C) the University of Chicago (U of Chicago) dataset (p<0.0001), microarray data presented by log2 transformed values.

To validate TOX3 expression in a large set of primary breast tumors, we analyzed three datasets, TCGA, the University of North Carolina (UNC), and University of Chicago (U of C) (Fig 2A–2C). The TCGA samples consist of normal breast tissues (n = 95), luminal A (n = 417), luminal B (n = 187), Her 2 amplified (n = 67) and basal-like (n = 141) breast tumors. Across all platforms, the data were consistent with greater expression of TOX3 in luminal and Her2-positive subtypes, compared to normal breast tissues and basal-like tumors. Together, our data demonstrated higher expression of TOX3 in luminal subtype cells/tumors compared to basal-like subtype or normal breast cells/tissues.

### eQTL analyses showed correlations between SNP variants and TOX3 expression

**Fig 3:**
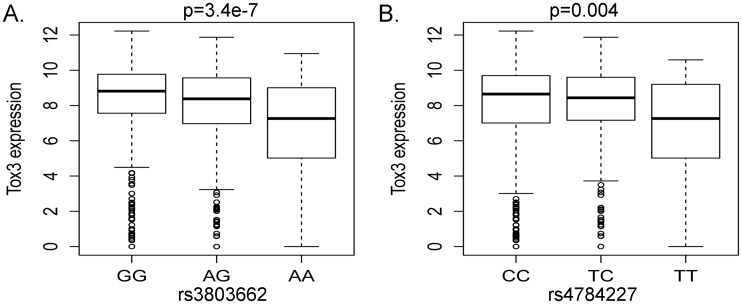
eQTL analyses between SNP genotypes and TOX3 expression. (A and B) eQTL analyses were performed on 345 SNPs located 500 kb upstream and 250 kb dowstream of the *TOX3* gene, using TCGA dataset. A significant association with TOX3 expression was observed in rs3803662 and rs4784227, a risk variant of breast cancer in European [7] and Asian women [8, 10], respectively.

To determine the effects of genetic variants associated with breast cancer on regulating TOX3 expression, we applied a linear regression model and conducted eQTL analyses on 345 SNPs genotyped 500 kb upstream and 250 kb downstream of the TOX3 gene, using TCGA dataset. We identified 77 SNPs associated with TOX3 gene expression which passed the multiple testing adjustments using a false discovery rate below 0.05 (S1 Table). A significant association with TOX3 expression was observed in rs3803662 (p = 3.4e-7), a risk variant of breast cancer in European women [7], with the minor A allele associated with lower TOX3 expression (Fig 3A). SNP rs4784227, a risk variant identified in Asian women [8,10], also showed a significant association with TOX3 expression (p = 0.004) with the minor T allele associated with increased risk of breast cancer and lower TOX3 expression (Fig 3B).

Our previous fine-mapping study in women of African ancestry revealed rs3104778 and rs3104788 as most-significant genotyped and imputed SNPs [11,12,13,14]. However, when we performed eQTL analyses of rs3112617 and rs3104788, we found no association between the variants and TOX3 expression (S1 Table). Together, our analyses confirmed rs3803662 and rs4784227 as eQTL SNPs significantly associated with TOX3 expression, whereas rs3112617 and rs3104788 have no influence on differential expression of TOX3 in luminal tumors.

### Luminal breast tumors exhibits lower methylation levels in the *TOX3* promoter

**Fig 4:**
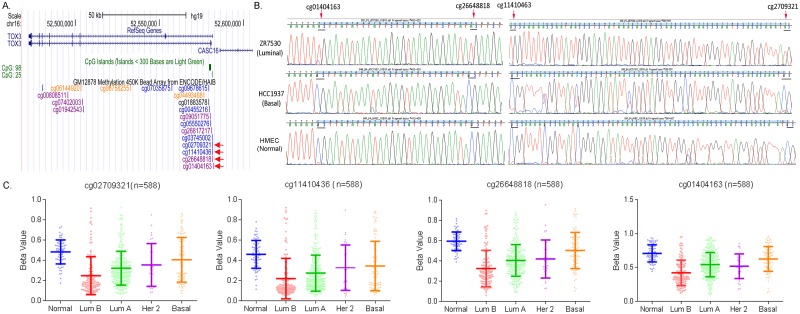
Subtype-specific methylation of the *TOX3* promoter in breast tumors. (A) UCSC genome browser shows location of CpG islands in the *TOX3* promoter at chromosome 16q12. Four CpG dinucleotides analyzed are indicated with red arrows. (B) Representative chromatographs in the *TOX3* promoter after bisulfite treatment revealed unmethylated cytosines in the *TOX3* promoter of ZR-75-30 (luminal subtype cells). DNA sequencing was performed on the reverse strand and four unmethylated CpG sites are underlined (AC, reverse complement of TG). In contrast, methylated cytosines (GC, reverse complement of CG) were detected in the promoter of HCC-1937 (basal-like subtype cells) and HMEC (primary epithelial normal cells) because methylation prohibited the effect of bisulfite treatment. (C) Methylation levels of four CpG dinucleotides in the promoter region of TOX3 were analyzed using the TCGA HumanMethylation450 Array data. One-way ANOVA and Tukey’s multiple comparison tests showed a significant difference in methylation levels of the CpG sites between normal breast tissues and luminal A or B breast tumors (p<0.0001) and between basal-like and luminal A or B breast tumors (p<0.01).

To determine if epigenetic regulation of the *TOX3* promoter contributes to the subtype-specific expression of TOX3, we performed bisulfite sequencing in breast cancer cell lines. UCSC genome browser view shows location of CpG islands in the promoter region at chromosome 16q12 (Fig 4A). Four CpG dinucleotides in the promoter region (cg02709321, cg11410436, cg26648818, and cg01404163) were sequenced after bisulfite conversion. A representative chromatograph of a normal epithelial (HMEC), a luminal (ZR7530), and a basal-like cell line (HCC-1937) showed clear difference in sequence reading with bisulfite treatment (Fig 4B). The four sites are all unmethylated in ZR7530 cells and converted into TG (AC in the reverse strand) with bisulfite treatment, whereas they are all methylated in HMEC and HCC-1937 cells (GC in the reverse strand).

**Fig 5:**
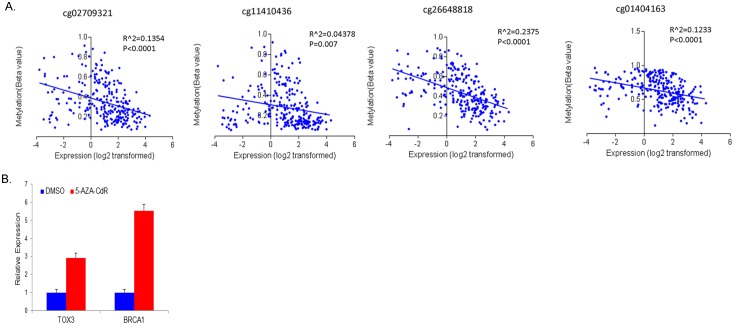
Expression of *TOX3* is correlated with the promoter methylation in breast tumors and increased by 5-aza-CdR treatment. (A) TCGA breast invasive carcinoma DNA methylation (Illumina Infinium HumanMethylation450 BeadChip, version 2014-05-02) and gene expression microarray (Agilent 244K, G4502A, version 2013-06-02) datasets were analyzed. Scatter plots show a significant inverse correlation between expression of *TOX3* and the promoter methylation at four CpG sites. (B) qRT-PCR results after UACC-3199 cells were treated for four days with 5-aza-CdR. Expression of TOX3 and BRCA1 were significantly increased after treatment.

To validate CpG site methylation of *TOX3* in a large set of breast tumors, we analyzed methylation levels of the four CpG dinucleotides using the TCGA HumanMethylation450 Array data. These results are expressed as beta values, which are continuous variables between 0 and 1. Hypomethylated regions have lower beta values while hypermethylated regions have higher beta values. Out of 838 patient samples with DNA methylation profile, we selected 588 samples for the analysis, after excluding samples from male patients, samples with no PAM50 subtype information, or samples showing discrepancy between PAM50 subtypes and pathological analyses (sample types). The included samples were comprised of normal breast tissues (n = 70), luminal B (n = 127), luminal A (n = 276), Her 2 (n = 30) and basal-like (n = 85) breast tumors. One-way ANOVA and Tukey’s multiple comparison tests at the four CpG islands showed significantly lower methylation in the CpG islands of the *TOX3* promoter in luminal A and B breast tumors compared to normal breast tissues and basal-like breast tumors (Fig 4C). Correlation analysis in the TCGA dataset showed a weak but statistically significant inverse correlation between methylation and expression levels of TOX3 at the four CpG sites (Fig 5A). These results indicate that promoter methylation of TOX3 may be a potential epigenetic modification resulting in the expression differences between the luminal and basal-like subtypes of breast cancer.

### Inhibition of methylation increased TOX3 expression in a basal-like cancer cell line

To determine if de-methylation of the promoter could increase expression of TOX3, we treated a basal-like breast cancer cell line (UACC-3199) with a DNA methylation inhibitor, 5-aza-2’-deoxycytidine (5-aza-CdR). We used *BRCA1* as an epigenetically regulated control gene, as we previously observed up-regulation of BRCA1 driven by de-methylation of the promoter in UACC-3199 cells [23]. As expected, BRCA1 expression was highly increased (a 5.52±0.36 fold increase) in 5-aza-CdR treated cells compared to vehicle alone (DMSO) (Fig 5B). Our data also showed a 2.93±0.27 fold increase in TOX3 expression in 5-aza-CdR treated cells compared to control, indicating that CpG methylation of the TOX3 promoter plays a significant role in the regulation of TOX3 expression in the breast cancer cell line.

### mQTL analysis showed a weak or no correlation between SNP variants and TOX3 methylation

**Fig 6:**
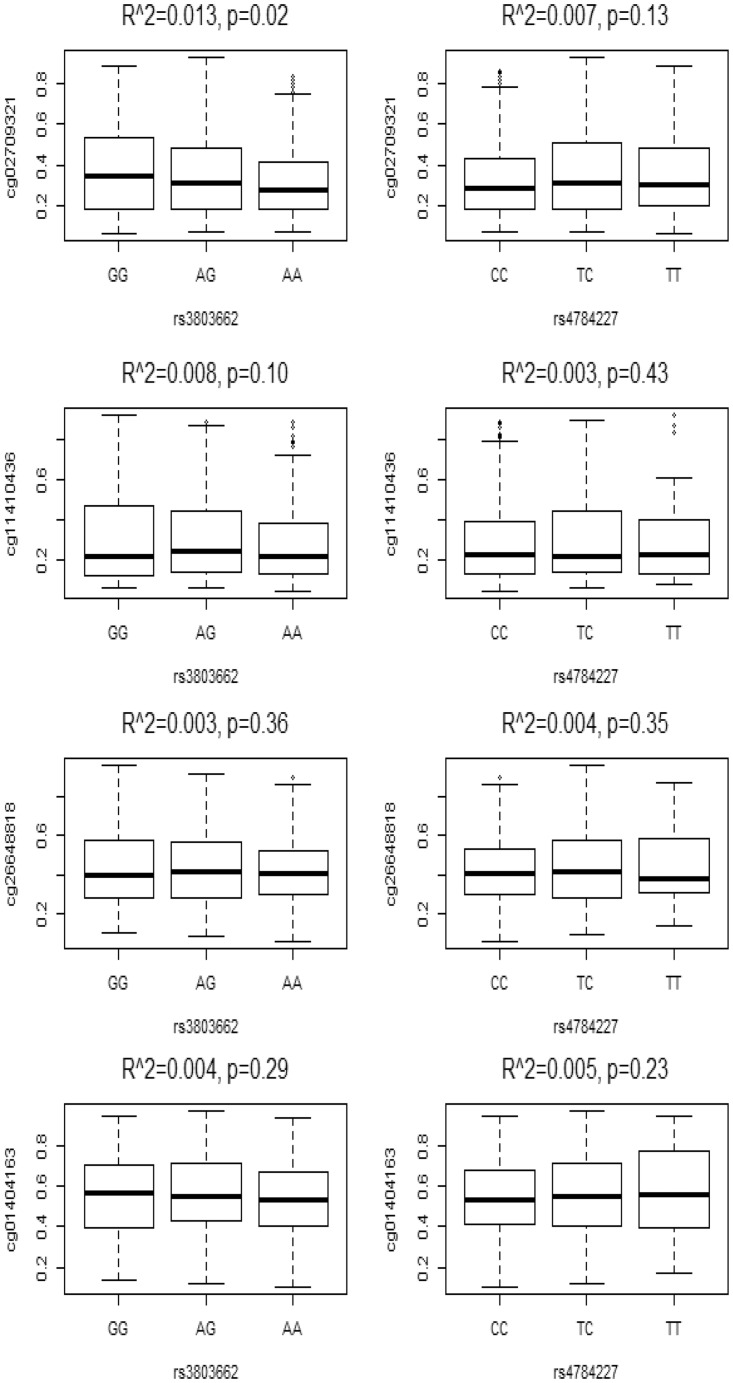
mQTL analyses between SNP genotypes and CpG site methylation of the TOX3 promoter. Linear regression model applied to mQTL analysis. Y-axis represents CpG sites (cg02709321, cg11410436, cg26648818, and cg01404163) and X-axis shows SNP variants (rs3803662 and rs4784227). R squared and p values shown on the top.

Because our data showed that both genetic and epigenetic factors contributed to the molecular subtype-specific expression of TOX3 in breast tumors, we tested for a relation between the two factors. We conducted methylation quantitative trait loci (mQTL) and examined a correlation between SNP variants (rs3803662 and rs4784227) and methylation of the four CpG dinucleotides (cg02709321, cg11410436, cg26648818, and cg01404163). We found that cg02709321 was weakly associated with rs3803662 but not with rs4784227 (Fig 6). None of other CpG sites (cg11410436, cg26648818, and cg01404163) were associated with either rs3803662 or rs4784227. Our data suggest that there is a weak or no correlation between the SNP genotypes and TOX3 promoter methylation.

### *TOX3* copy number does not correlate with expression

**Fig 7:**
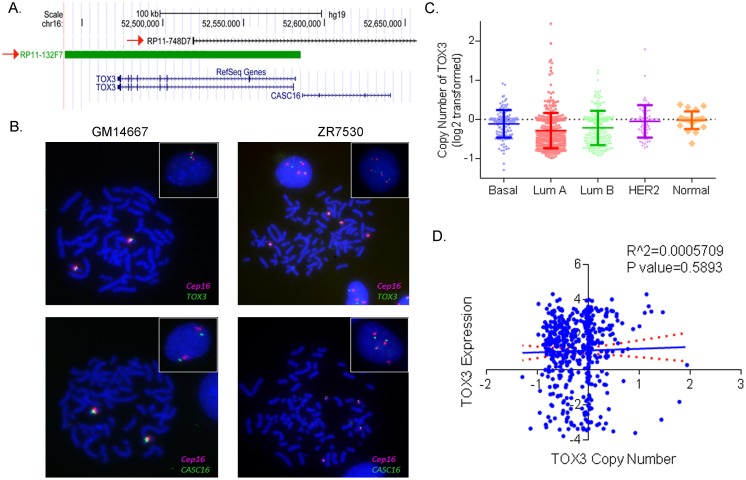
Copy number of *TOX3* in breast tumors. (A) Genomic positions of BAC RP11-132F7 and RP11-748D7 at chromosome 16q12. These clones were selected for homebrewed TOX3 and CASC16 FISH probes, respectively. (B) Representative FISH photomicrographs of *TOX3*:CEP16 and *CASC16*:CEP16 in GM14667 (control normal lymphocytes) and ZR-75-30 (luminal) cell lines. Cells were counterstained with DAPI (blue), while *TOX3 or CASC16* is localized by green fluorescent signal, and *CEP16* is localized by a red fluorescent signal. Metaphase and interphase (insert) cells are shown. These results are summarized in S2 Table. (C) TCGA copy number data analysis shows copy number of *TOX3* in breast cancer subtypes. (D) Scatter plot shows no correlation between copy number and expression of *TOX3* in the breast tumors.

To determine if subtype-specific expression of TOX3/CASC16 is due to deletions or amplifications of the genes, we performed FISH using two home-brewed probes, RP11-132F7 and RP11-748D7, which contain *TOX3* and *CASC16*, respectively (Fig 7A). The chromosome 16-specific DNA repeat probe (*CEP16*) was used as a control [24] and the probe hybridization efficiency was validated on the normal lymphocyte cell line GM14667. The results showed a normal pattern of two copies of each signal with *TOX3*:*CEP16* ratio of 1.0 (Fig 7B). The normal breast epithelium cell line HMEC also exhibited a normal pattern of 2:2 (*TOX3*:*CEP16*, seen in 88% of cells) (S2 Table). Gene copy number of *TOX3* were examined in six breast cancer cell lines, including four luminal breast cancer cells (ZR-75-30, MDA-MB-175VII, HCC-202, and T-47D) and two basal-like breast cancer cells (HCC-70 and HCC-1500). Breast cancer cells displayed some degree of abnormal signal patterns including polysomy (ZR-75-30, HCC-70, and MDA-MB-175VII), but no actual gene amplification or deletion was observed (S2 Table).

We next examined *TOX3* CNV in a large set of breast tumors using TCGA GISTIC2 CNV dataset and demonstrated similar levels of copy numbers across different subtypes with slightly lower levels in luminal tumors (Fig 7C). No correlation was observed between the copy number and expression of *TOX3* (P = 0.58). The data suggested no influence of CNV on regulating gene expression of *TOX3*. Taken together, our data indicate the increased expression of TOX3 in luminal tumors is associated with CpG island methylation as well as SNP genotypes, but not with copy number alterations.

## Discussion

In this study, we investigated both genetic and epigenetic factors contributing to TOX3 expression in breast tumors and cell lines. We have confirmed that TOX3 expression is highly up-regulated in luminal breast cancer compared to normal breast tissues or basal-like tumors. The molecular subtype-specific expression of TOX3 in breast tumors is significantly associated with epigenetic modifications at CpG islands of the promoter. Germline genotypes also contribute to TOX3 expression with a significant association of rs3803662 and rs4784227 with TOX3 expression in breast tumors. In contrast, no correlation between CNV and expression of *TOX3* gene was observed.

The SNPs at 16q12, close to the *TOX3* and *CASC16* genes, represent one of the susceptibility loci identified by GWAS, showing strong evidence for breast cancer association across various populations. A functional study following GWAS showed that the risk allele of rs3803662 and the mRNA level of TOX3 predicted adverse outcomes for breast cancer patients [19]. The minor allele of SNP rs3803662 has been shown to correlate with increased breast cancer risk and with lower expression of TOX3. The SNP rs4784227, a risk variant for breast cancer in Asian women, was also reported to alter TOX3 expression by disrupting enhancer function through FOXA1 affinity modulation [16]. Through TCGA dataset, we confirmed that rs3803662 and rs4784227 are associated with TOX3 expression in breast tumors. The association of rs380662 with TOX3 expression has been previously observed, further demonstrating the utility of publicly available data [25,26]. Interestingly, the risk variants in women of African ancestry (rs3103104788 or rs3112617) did not show an association with TOX3 expression in breast tumors, suggesting the effects of risk variants on regulating TOX3 expression might be risk allele-specific or ethnicity-specific.

In addition to the previous functional study of risk variants, we investigated epigenetic regulation of TOX3 expression on breast tumors *in silico*, revealing an inverse correlation between subtype-specific expression of TOX3 and CpG site methylation of the promoter. Transcription factors associated with ER (i.e. FOXA1) are also likely to modulate the *TOX3* promoter activity, based on the previous observation that SNP rs4784227 alter TOX3 expression by disrupting enhancer function through FOXA1 affinity modulation [16]. This suggests that methylation of TOX3 may be one of several factors involved in the regulation of TOX3 in breast cancer.

It is possible that SNPs rs3803662 and rs4784227 modulate CpG island methylation and thus contribute to subtype-specific expression of TOX3 in breast cancer. We tested the possibility by performing mQTL between the SNP variants and TOX3 promoter methylation in breast tumors. mQTL analyses showed a weak or no correlation of rs3803662 or rs4784227 with four CpG sites in breast tumors (cg02709321, cg11410436, cg26648818, and cg01404163). Our data suggest that the genetic and epigenetic regulations might be independent and perhaps represent no related events in regulating TOX3 expression.

Functionally, TOX3 belongs to the high-mobility-group (HMG)-box family of proteins that modify chromatin structure [27]. It is expressed mainly in epithelial cells and targets both antiapoptotic and proapoptotic transcripts [28]. High expression of TOX3 is correlated with ER and progesterone receptor expression as well as positive lymph nodes [19]. Patients with ER positive tumors and high levels of TOX3 mRNA had shorter overall- and distant metastasis free-survival. Although more mechanical studies are required to determine the regulation of TOX3 expression by epigenetic modifiers, our findings on the upregulation of TOX3 by cytosine methylation raise the possibility of new epigenetic biomarkers for prognosis of aggressive ER positive breast tumors.

## Materials and Methods

### Cell culture and total RNA and DNA isolation

Human mammary epithelial (HMEC) primary cells were purchased from Lonza and UACC3199 cells were obtained from University of Arizona Cancer Center. Other breast cancer cells were obtained from the American Type Culture Collection (Manassas, VA). Cells were tested negative for mycoplasma contamination and validated for species and unique DNA profile using short tandem repeat analysis by the provider or us. Twenty two breast cancer cell lines were cultured and maintained in the specified media. Breast epithelium cell lines 184A1 and 184B5 as well as primary cell line HMEC were cultured in MEGM medium (Lonza CC-3150). Breast cancer cell lines CAMA1 and MCF7 (with additional 0.01mg/ml bovine insulin) were cultured in MEM medium (Corning 10–010) with 10% FBS. HS578T was cultured in DMEM medium (Gibco 11995–065) with 10% FBS and 0.01mg/ml bovine insulin. All other cell lines were cultured in RPMI 1640 medium (Gibco 11875–093) with 10% FBS and 1% HEPES. Total RNA and DNA were isolated from cell lines using the RNeasy Mini Kit (Qiagen, Montgomery, MD, USA) and GentraPuregene Cell Kit (Qiagen, Montgomery, MD, USA), respectively. The integrity of RNAs was validated by bio-analyzer at the University of Chicago Genomics Core Facility. RNAs with minimum RNA integrity number of 8 were applied to cDNA synthesis.

### qRT-PCR

cDNA was generated by SuperScript III First-Strand synthesis Super Mix for quantitative real-time polymerase chain reaction (qRT-PCR)(Life Technologies, Carlsbad, CA, USA). The assessment of mRNA levels was performed using a 7900HT Fast Real-Time PCR system (Life Technologies, Carlsbad, CA, USA) using Power SYBR Green PCR Master Mix (Life Technologies) or TaqMan Gene Expression Master Mix (Life Technologies). All real-time PCRs were performed in four replications, and the fold change in expression of mRNAs was calculated using the ΔΔCt method, with rRNA18Sas an internal control.

### Fluorescent In Situ Hybridization (FISH) with home-brewed probes

To evaluate copy number of *TOX3* and *CASC16* in breast cancer cell lines, we carried out FISH using two home-brewed probes of RP11-132F7 and RP11-748D7. These BAC clones were selected from the UCSC genome browser as it contains the region of 16q12 that covers *TOX3* and *CASC16* gene respectively (http://genome.ucsc.edu/). The probes were prepared using the Abbott Nick Translation Kit (Abbott Laboratories, Abbott Park, IL) and labeled with Spectrum Green. Cell lines were scored and digital images were obtained using the Zeiss AXIO IMAGER Z2 microscope and Zeiss AxioCamMRm Rev 3 Monochromatic Camera. For each cell line, signals were counted in 60 metaphase and interphase cells with well-defined nuclei and averaged. The ratio of the *TOX3* and *CASC16* signal to *CEP16* signal and percentage of each signal pattern was used to determine copy number in each cell line. Polysomy was defined as greater than three copies of a chromosome. The ratio ranges for deletion and amplification of *TOX3*:*CEP16* and CASC16:CEP16 are as follows: deletion is < 0.8, >1 and < 2 is gain, and >2 is amplification.

### Bisulfite sequencing

Four CpG islands in the *TOX3* promoter were selected from TCGA data. Bisulfite modification of 0.5μg of DNA from breast cancer cell lines was carried out with the EZ DNA Methylation-Gold™ kit (Zymo Research, Irvine, CA) according to the manufacturer’s protocol. The PCR reactions consisted of the bisulfite-modified DNA and ZymoTaq (Zymo Research, Irvine, CA) with forward (5’-TTAGGTTTTGGGTTAGTAAGGTGTG-3’) and reverse (5’-ACCCCCTTCCTTCTTCATAAATAC-3’) primers, at an annealing temperature optimized for each primer. The PCR products were run on a gel to confirm specificity. After cleaning PCR products with Exonuclease I (New England BioLabsInc, Ipswich, MA) and Shrimp Alkaline Phosphatase (Affymetrix, Santa Clara, CA), Sanger sequencing was performed at the University of Chicago Comprehensive Cancer Center DNA Sequencing and Genotyping Facility. Chromatograms of DNA sequencing were read on Chromas Lite 2.1.1 (Technelysium, South Brisbane, Australia).

### Treatment of 5-Aza-2’-Deoxycytidine

UACC-3199 was seeded at a density of 3 X 10^5^ per well of 6 well plate. One day after plating, cells were treated with 5-Aza-2’-Deoxycytidine (5-aza-CdR) at 8uM dose in DMSO and incubated for four days. Control sample was treated with same amount of DMSO. Nucleic acids were extracted using AllPrep DNA/RNA/Protein Mini Kit (Qiagen, Montgomery, MD) and subjected to qRT-PCR analysis.

### TCGA data and statistical analysis

TCGA breast invasive carcinoma CNV (GISTIC2 method, version 2014-05-09), RNA-seq (Illumina HiseqV2, version 2014-05-09), gene expression microarray (Agilent 244K, G4502A, version 2013-06-02) and DNA methylation (Illumina Infinium HumanMethylation450 BeadChip, version 2014-05-02) datasets were extracted from UCSC Cancer Browser (https://genome-cancer.ucsc.edu/), along with the clinical-pathological phenotypes. In the analyses, we excluded samples from male patients, samples with no PAM50 subtype information, or samples showing discrepancy between PAM50 subtypes and pathological analyses (sample types). We performed the one-way ANOVA and Tukey’s multiple comparison tests to compare the methylation level across the breast tumor subtype groups. Statistical analysis was conducted and plots were generated by GraphPad Prism 6.0 (GraphPad Software, Inc., La Jolla, CA), R or Stata.

## Supporting Information

S1 Table eQTL results. (XLSX)

S2 Table Gene copy number of *TOX3*. (DOC)
