# Supplementary material for: Genetic and Epigenetic Regulation of TOX3 Expression in Breast Cancer
Source: PLoS One. 2016 Nov 2;11(11):e0165559. doi: 10.1371/journal.pone.0165559 (PMC5091860; doi:10.1371/journal.pone.0165559)
Supplement: S2 Table — (DOC) [file pone.0165559.s002.doc]

**S2 Table.** Gene Copy Number (GCN) of *TOX3*

| **Cell Line** | **# of cells scored** | **TOX3 GCN^1^** | **CEP16 GCN^2^** | **TOX3:CEP16^3^** | **Representative clone(s) (TOX3:CEP16,%cells)** | **TOX3 Amplification** | **Interpretation^4^** |
| --- | --- | --- | --- | --- | --- | --- | --- |
| HMEC | 60 | 2.0 | 2.2 | 1.0 | 2:2(88%) | No | Normal |
| ZR7530 | 60 | 4.1 | 3.9 | 1.0 | 4:4(77%) | No | Polysomy |
| MDAMB175VII | 60 | 3.1 | 3.1 | 1.0 | 3:3(73%) | No | Polysomy |
| HCC202 | 60 | 2.0 | 2.6 | 0.8 | 2:3(55%) | No | Abnormal, heterogenity |
| T47D | 60 | 2.0 | 2.8 | 0.7 | 2:3(83%) | No | Abnormal |
| HCC70 | 60 | 6.4 | 6.3 | 1.0 | 6:6(63%) | No | Polysomy |
| HCC1500 | 60 | 2.0 | 2.0 | 1.0 | 2:2(98%) | No | Normal |

^1^Mean copy number of gene per cell

^2^mean copy number of centromere enumeration probe (CEP) per cell;

^3^mean gene to CEP ratio;

^4^GCN classification; polysomy, ≥ three copies in >90% of cells
